# Proposal of a modified tip apex distance for prediction of lag screw cut-out in trochanteric hip fractures

**DOI:** 10.1051/sicotj/2023026

**Published:** 2023-09-20

**Authors:** Amr Selim, Nawfal Al-Hadithy, Nader M. Diab, Abdulla Mohamed Ahmed, Khaled Fawzy Abdel Kader, Mohamed Hegazy, Hazem Abdel Azeem, Ahmed Samir Barakat

**Affiliations:** 1 Cairo University Hospitals Cairo 11562 Egypt; 2 Oswestry/Stoke, The Shrewsbury and Telford Trust TF1 6TF UK; 3 Hillingdon Hospital Uxbridge UB8 3NN UK

**Keywords:** Trochanteric fractures, DHS, Lag screw, Tip Apex Distance, Fixation failure, Cut-out

## Abstract

*Introduction*: Lag screw cut-out is a serious complication of dynamic hip screw fixation of trochanteric hip fractures. The lag screw position has been acknowledged as one of the important factors affecting the lag screw cut-out. We propose a modification of the Tip Apex Distance (TAD) and hypothesise that it could improve the reliability of predicting lag screws cut-out in these injuries. *Materials and Methods*: A retrospective study was conducted for hip fracture entries in the period from Jan 2018 to July 2022. A hundred and nine patients were suitable for the final analysis. The modified TAD was measured in millimetres based on the sum of the traditional TAD in the lateral view and the net value of two distances in the AP view, the first distance is from the tip of the lag screw to the opposite point on the femoral head along the axis of the lag screw while the second distance is from that point to the femoral head apex. The first distance is a positive value, whereas the second distance is positive if the lag screw is superior and negative if inferior. A receiver operating characteristic curve was used to evaluate the reliability of the different parameters assessing the lag screw position within the femoral head. *Results*: Reduction quality, fracture pattern as per the AO/OTA classification, TAD, Calcar Referenced TAD, Axis Blade Angle, Parker’s ration in the AP view, Cleveland Zone 1, and modified TAD were statistically associated with lag screw cut-out. Among the tested parameters, the modified TAD had 90.1% sensitivity and 90.9% specificity for lag screw cut-out at a cut-off value of 25 mm with a *P*-value < 0.001. *Conclusion*: The modified TAD had the highest reliability in the prediction of lag screw cut-out. A value ≤ 25 mm could potentially protect against lag screw cut-out in trochanteric hip fractures.

## Introduction

Implant failure following surgical treatment of trochanteric hip fractures is a devastating problem which leads to a significant increase in morbidity and mortality [1]. Several factors have been identified to affect fracture stability and implant failure [2–6]. These include patient age, bone quality, fracture pattern, quality of fracture reduction, and position of the lag screw within the femoral head [2–6].

Tip Apex Distance (TAD) is one of the important parameters predicting lag screw cut-out and subsequent implant failure [7, 8]. Biomechanical and clinical studies reported the optimal position of the lag screw to be central or inferior in the anteroposterior (AP) view and central in the lateral (LAT) view [7–13]. While this safe position of the lag screw in the femoral head has been verified, the TAD does not account for the lag screw direction within the femoral head, therefore inferior and superior lag screws could have the same value despite having a different biomechanical stiffness and cut-out probability [9, 12]. Several parameters were developed to guide lag screw placement such as Cleveland Zones, Parker’s ration, and Calcar-referenced Tip Apex Distance (Cal TAD), however, none of them reflects the lag screw insertion depth and direction simultaneously [9–11]. Recently, Axis Blade Angle (ABA) has been introduced to solve the direction issue, yet this parameter does not consider the lag screw insertion depth [12].

The rationale of this study is to propose a modification of the TAD that could give more precise information about the lag screw insertion depth as well as direction, aiming to improve the reliability of predicting lag screw cut-out in these patients.

## Materials and methods

The current study was conducted in a retrospective manner. The study was registered with the Research Ethical Committee with a reference number “MD-277-2020”.

*The inclusion criteria were:*

- Elderly patients > 60 years old.
- Pertrochanteric fracture; 31 A1, and A2 fractures according to the AO/OTA classification, underwent Dynamic Hip Screw (DHS) fixation.
- Completion of 12 months follow up.
- Standard Hip radiographs.

*The exclusion criteria were:*

- Patients with neurological problems.
- Loss of follow-up information.

### Sample size

The sample size was calculated using the Power Analysis and Sample Size (PASS) software program version 11.0.10 (NCSS LLC, Kaysville, UT) with 0.05 alpha error, confidence interval of 0.95 and study power of 0.90. The minimum required number of patients was 102. Hip fracture entries for the period from Jan 2018 to Jul 2022 were reviewed by generating a query from the hospital database. The initial search generated 921 entries with hip fractures, of which 598 were intracapsular fractures, and 323 extracapsular fractures. Of the extracapsular fractures, 147 were treated with cepahlomedullary nails (CMN) and 176 had DHS. Thirty six patients had non-standard intra-operative radiographs, while 31 patients had early mortality or lost follow-up, hence were excluded. This left 109 patients suitable for the final analysis.

### Variables

The recorded data included age, gender, pre-injury mobility status, comorbidities, abbreviated mental test score (AMTS), and time to operation.

Pre-operative X-rays were analysed for the fracture type according to AO/OTA classification [4], reduction quality and degree of osteoporosis using Singh Osteoporosis Index (SOI) of the contralateral hip radiographs [8, 14]. Intra-operative radiographs were analysed for the following parameters: TAD, Cal-TAD, Parker’s ration in AP and LAT views, Cleveland Zone, ABA, and the modified TAD [8–12]. Calibration was performed using the lag screw diameter in the intra-operative X-rays as a reference value (12.5 mm).

**Figure 1:**
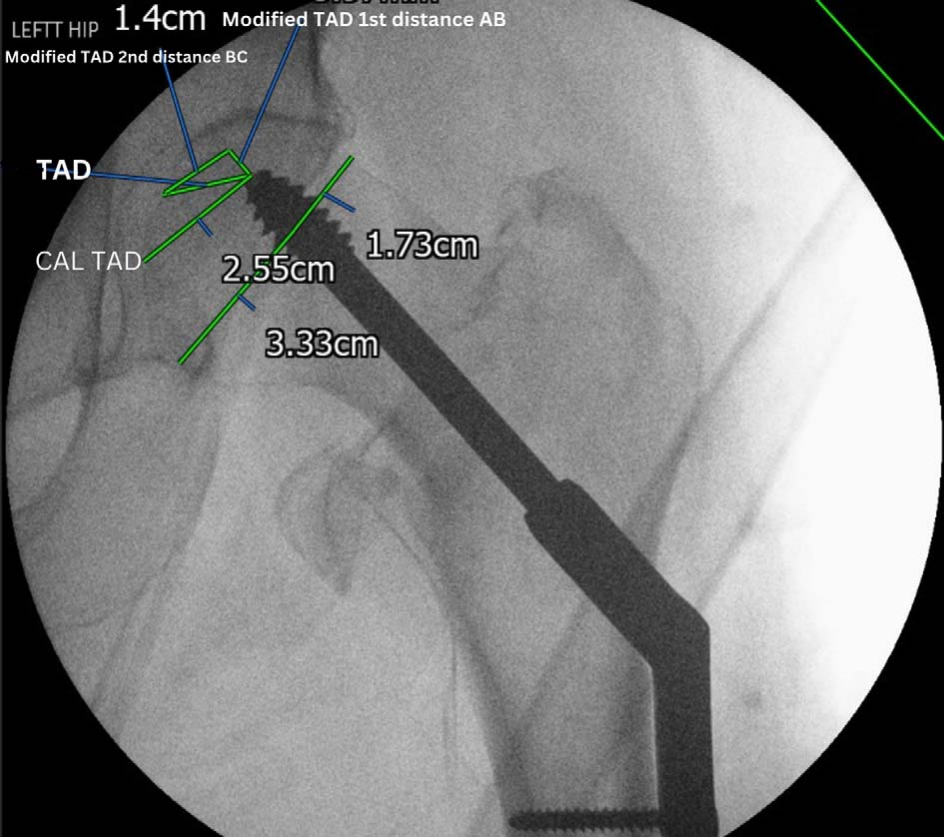
Intraoperative X-ray showing the technique used to measure TAD, Cal TAD, and modified TAD.

Reduction quality in the X-rays was classified into three grades; good, acceptable, and poor, based on a method developed by Baumgaertner et al. [8]. The TAD, Cal TAD, Parker’s ration, Cleveland zone, ABA, and the modified TAD were recorded by 2 candidates and the mean value was considered (Figure 1).

**Figure 2:**
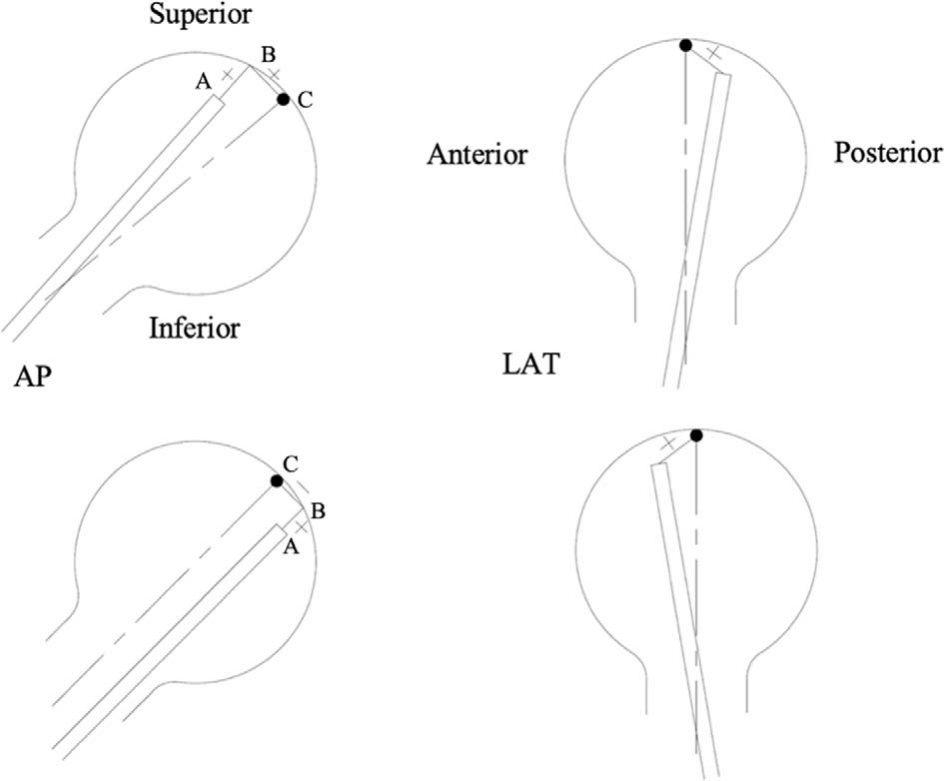
Diagram showing the modified TAD measurement in the AP (left) and LAT (right) views. The top left shows the calculation for superior lag screws and the bottom left shows the calculation for inferior lag screws.

The modified TAD was measured in millimetres (mm) based on the net value of two distances in the AP view added to the traditional TAD in the LAT view. In the AP view, the first distance is from the tip of the lag screw (point A) to the opposite point on the femoral head along the axis of the lag screw (point B). The second distance is from point B to the femoral head apex (point C). The first distance (AB) is always a positive value, but the second distance (BC) value depends on the location of the lag screw within the femoral head in relation to the head central axis. If the lag screw is inferior, then the second distance (BC) is a negative value. If the lag screw is superior, the second distance (BC) is a positive value. If the lag screw is central within the femoral head along the central axis of the neck, there is only one distance from the tip of the lag screw to the femoral head apex. This means if the lag screw is inferior within the femoral head, the net value of the novel parameter would be less than another screw which has the same distance from the femoral head apex yet is superior. This has been depicted in Figure 2.

### Follow up

Two candidates reviewed the post-operative x-rays at 6 weeks, 3, 6 and 12 months. Mechanical complications were recorded, and any discrepancy was resolved by consensus amongst all authors. The collected data were analysed to investigate the sensitivity and specificity of the studied parameters in detecting lag screw cut-out.

### Statistical methods

The statistical work was done by a statistician. Data were coded and entered using the statistical package for the Social Sciences (SPSS) version 28 (IBM Corp., Armonk, NY, USA). Data were summarized using mean, standard deviation, median, minimum and maximum in quantitative data, and using frequency (count) and relative frequency (percentage) for categorical data. Comparisons between quantitative variables were done using the non-parametric Mann–Whitney test [15]. For comparing categorical data, Chi-square (χ^2^) test was performed. An exact test was used when the expected frequency is less than 5 [16]. Receiver operating characteristic (ROC) curve was constructed with the area under curve analysis performed to detect the best cutoff value of different parameters for the detection of complications. *P*-values less than 0.05 were considered statistically significant [17].

## Results

1. Patients’ demographics
  - Age distribution
    Patients had a mean age of 81.61 years with a standard deviation (SD) ± 9.02 years. The Median was 83.00. There was no statistically significant relation between age and lag screw cut-out with a *P*-value of 0.067.
  - Gender distribution
    There were 18.3% males and 81.7% females in the study group. There was no statistically significant relation between gender and lag screw cut-out.
  - Pre-injury Mobility status
    45.9% of patients used to mobilise independently prior to the injury, 27.5% using one aid, 11.9% using a frame, and 14.7% only indoors. There was no statistically significant relation between the mobility status and lag screw cut-out with a slightly higher rate in the frame group.
  - Co-morbidities
    Hypertension was prevalent in 47.7% of patients, cardiac problems in 31.2%, diabetes mellitus in 17.4%, and dementia in 22.9% of patients. The presence of diabetes mellitus (DM) has a statistically significant relation with mortality *P*-value 0.044.
  - AMTS at presentation
    Patients with AMTS ≤ 7/10 at presentation represented 29.4% of the study group, while 48% had AMTS 10/10.
2. Peri-operative outcomes
  - Time to operation
    The time between diagnosis and operation was ≤ 24 hours (h) in 80.7% of patients, 48 h in 13.8%, and 72 h in 5.5% of patients. Longer time to operation between 24 and 48 h was not associated with a statistically significant increase in mortality (*P*-value 0.152), however, 50% of patients who had their operation at 72 h died in the early post-op period. The reasons reported for the delay in the surgical intervention were awaiting theatre space, awaiting medical optimisation, or delay because of the COVID pandemic.
  - Fracture pattern
    The fracture pattern distribution in the study group as per the AO/OTA fracture classification is shown in Table 1. There was a statistically significant relation between the fracture pattern and lag screw cut-out with a higher cut-out rate in A2.2 and A2.3 patterns.
      **Table 1:** Fracture patterns in the study group and its relation to lag screw cut-out.

		Total		Lag screw cut-out				
				Y		N		
		Count	%	Count	%	Count	%	*P*-value
Fracture pattern (AO/OTA classification)	A1.2	56	51.4	0	0.0	56	100.0	<0.001
	A1.3	28	25.7	3	10.7	25	89.3	
	A2.2	18	16.5	6	33.3	12	66.6	
	A2.3	7	6.4	4	57.1	3	42.9
  - Reduction quality
    Table 2 shows the quality of reduction in the study group as per the method proposed by Baumgaertner et al. [8]. There was a statistically significant relation between reduction quality and lag screw cut-out.
      **Table 2:** Reduction quality in the study group and its relation to lag screw cut-out.

		Total (%)	Lag screw cut-out		
			Y (%)	N (%)	*P*-value
Reduction quality	Good	53.2	1.7	98.3	<0.001
	Acceptable	42.2	10.9	89.1	
	Poor	4.6	80.0	20.0
  - Singh Osteoporosis index
    79.8% of patients in the study group were in groups 3 and 4. There was no statistically significant relation between SOI and lag screw cut-out within the study group.
3. Lag screw parameters
  There was a statistically significant relation between the following parameters: TAD, Cal TAD, and the modified TAD with lag screw cut-out with a *P*-value < 0.001. Parker’s ration in the AP, Cleveland Zone 1, and ABA were associated with lag screw cut-out with a *P*-value of 0.002, 0.004 and 0.045 respectively. Parker’s ratio in the lateral view did not show a statistically significant relation with lag screw cut-out. Table 3 shows AUC, cut-off, sensitivity and specificity of the different parameters in predicting lag screw cut-out. The modified TAD showed the highest combined sensitivity 90.1% and specificity 90.9% for detecting lag screw cut-out. The reliability of the modified TAD has been tested at different values using an ROC curve and the best cut-off was at 25 mm.
    **Table 3:** Reliability of different parameters in predicting lag screw cut-out.

	Area under the curve	*P*-value	95% confidence interval				
			Lower Bound	Upper Bound	Cut-off	Sensitivity %	Specificity %
TAD (mm)	0.923	<0.001	0.825	0.911	25	89.3	88.5
CalTAD (mm)	0.938	<0.001	0.943	1.003	25	90.4	73
Parker’s ratio AP (%)	0.830	0.001	0.636	1.024	64.5	75	98.9
Modified TAD (mm)	0.934	<0.001	0.855	1.013	25	90.1	90.9
4. Mechanical outcomes
  Lag screw cut-out occurred in 13 (11.9%) patients, 10 of them were recognised in the first 3 months postoperatively. Other surgical complications occurred in 4 (3.7%) patients. This included femoral head AVN 2.7% (3 patients) and medialisation with screw breakage 0.9% (1 patient). Revision surgery was done in 13 patients (11.9%) in total. Revision surgery was in the form of total hip replacement 8.4% (9 patients) and proximal femoral replacement 3.6% (4 Patients).

## Discussion

Lag screw position within the femoral head is one of the important factors influencing lag screw failure in trochanteric hip fractures [13]. Several parameters were developed to guide lag screw insertion within the femoral head as shown in Table 4 [8–12], though none of them considers both the lag screw insertion depth and direction. We proposed a modification of the TAD to assess the lag screw position and conducted this study to evaluate its reliability, along with other parameters, to predict lag screw cut-out. The main findings of our study prove a statistically significant relationship between lag screw cut-out and the following parameters: TAD, Cal TAD, Parker’s ratio in the AP view, Cleveland zone 1, ABA and the modified TAD. The modified TAD showed the highest reliability in predicting lag screw cut-out at a cut-off value of 25 mm. Poor reduction quality and more complex fracture pattern as per AO/OTA classification were two independent factors associated with lag screw cut-out.

**Table 4:** Recommendations from key studies to protect against lag screw cut-out.

Study	Parameter	Recommendation to protect against cut-out
Cleveland et al. 1959	Cleveland 9 zones system	Safe zones 5, 6, 8, 9
Parker et al. 1992	Parker’s Ration	A ration of 45% in the AP and LAT
Baumgaertner et al. 1995	TAD	TAD < 25 mm
Kuzyk et al. 2012	CAL TAD	Smaller CAL TAD (No value was given)
Mao et al. 2021	ABA	ABA > −10
Current study 2023	Modified TAD	Modified TAD ≤ 25 mm

Although the modified TAD had about 2% better sensitivity and specificity than the TAD in this study, we believe a bigger cohort of patients is required to conclude more precise results. We also suggest that future research, involving a greater number of lag screw cut-outs and fractures fixed with CMN, shall be conducted to provide robust conclusions.

Baumgaertner et al. populated the idea of TAD in the nineties as a predictive measure of lag screw cut-out. In their study of 198 cases, no complications of lag screw cut-out were noted in any patient with a TAD less than 25 mm. However, in patients with a TAD of more than 30 mm, 27% of patients experienced lag screw cut-out. They concluded that TAD of more than 30 mm increased the risk of cut-out and should be avoided [8]. However, not all patients with TAD values more than 30 mm had lag screw cut-out, thus other factors needed to be evaluated. Further reports have confirmed the relation between TAD and lag screw failure [7, 12, 13].

In an interesting biomechanical study, Kane et al. evaluated the mechanical stability of the central lag screw position in the AP and LAT views in comparison with AP inferior and LAT central lag screw positions in instrumented models. There was no difference in the biomechanical strength between both groups. They suggested similar clinical results in patients treated with AP inferior LAT centre lag screw construct, although they have a TAD of more than 25 mm [13].

In an attempt to provide an objective assessment of the lag screw direction and evaluate its relation to the cut-out, Mao et al. proposed a new parameter, ABA. They concluded a threshold value for lag screw cut-out of −10 degrees. However, their method does not account for lag screw insertion depth, therefore they suggested using both ABA and TAD or Cal TAD as predictive methods for lag screw cut-out [12].

To summarise, the TAD takes into consideration the lag screw insertion depth. However, the TAD cannot solve the direction issue, therefore two lag screws with the same TAD value could have distinctly different positions within the femoral head [18]. The Cal TAD moves the original reference point inferiorly to be close to the femoral calcar in AP view, but this modification has not attained universal agreement [19]. Parker’s ration has not been reported as a reliable predictor of lag screw cut-out in several studies, in addition to being a difficult method [11, 12]. Cleveland zones system only provides a subjective assessment of the lag screw position within the femoral head [10], while the ABA does provide a more objective assessment of the lag screw direction, but without consideration of the lag screw insertion depth [12]. Therefore, none of the existing parameters provides an objective assessment of the screw insertion depth and direction at the same time.

The theoretical advantage of the modified TAD is that it combines the lag screw insertion depth and direction in one method. Superior and inferior screws would not have the same value if they have the same insertion depth contrary to the TAD. It also retains the advantage of a central lag screw in the AP plane. The Cal TAD theoretically gives an advantage to the inferior lag screws over the central ones which have not been proved correct in many studies [6, 19]. The TAD modification was only proposed in the AP view because posterior or anterior lag screws in the LAT plane did not show similar or better biomechanical stability compared with central screws [12].

The strength of the current study, besides proposing a modification of the TAD, comes from several aspects. This study has particularly focused on trochanteric fractures in the elderly >60 years old as this cohort of patients has a different bone quality compared with the younger population, which explains why most patients had SOI grade 3 or 4. The follow-up time was 12 months, longer than other similar studies. The inclusion of trochanteric fracture 31A1 and 31A2 fractures and the exclusion of other patterns such as 31B and 31A3 improves the reliability of the result because these fractures have other independent risk factors for lag screw cut-out.

## Conclusion

The modified TAD shows the highest combined sensitivity (90.1%) and specificity (90.9%) compared with other parameters predicting lag screw failure in patients with trochanteric hip fractures. Modified TAD value ≤ 25 mm reflects a potentially stable lag screw position within the femoral head with a low possibility of lag screw cut-out. Hence, we recommend the use of the modified TAD as a predictive measure for lag screw cut-out.
